# Cost-Effectiveness of Mobile Health–Based Integrated Care for Atrial Fibrillation: Model Development and Data Analysis

**DOI:** 10.2196/29408

**Published:** 2022-04-19

**Authors:** Xueyan Luo, Wei Xu, Wai-Kit Ming, Xinchan Jiang, Quan Yuan, Han Lai, Chunji Huang, Xiaoni Zhong

**Affiliations:** 1 School of Public Health and Management Chongqing Medical University Chongqing China; 2 School of Biological and Chemical Engineering Chongqing University of Education Chongqing China; 3 Research Center for Medicine and Social Development Chongqing Medical University Chongqing China; 4 School of International Pharmaceutical Business China Pharmaceutical University Nanjin China; 5 Department of Infectious Diseases and Public Health City University of Hong Kong Hong Kong Hong Kong; 6 School of Pharmacy The Chinese University of Hong Kong Hong Kong Hong Kong; 7 Chong Qing Pharmaceutical Group Co Ltd Chongqing China; 8 School of Basic Medical Science Army Medical University Chongqing China

**Keywords:** mobile health, integrated care, ABC pathway, atrial fibrillation, model-based, cost-effectiveness, health economic evaluation

## Abstract

**Background:**

Mobile health (mHealth) technology is increasingly used in disease management. Using mHealth tools to integrate and streamline care has improved clinical outcomes of patients with atrial fibrillation (AF).

**Objective:**

The aim of this study was to investigate the potential clinical and health economic outcomes of mHealth-based integrated care for AF from the perspective of a public health care provider in China.

**Methods:**

A Markov model was designed to compare outcomes of mHealth-based care and usual care in a hypothetical cohort of patients with AF in China. The time horizon was 30 years with monthly cycles. Model outcomes measured were direct medical cost, quality-adjusted life years (QALYs), and incremental cost-effectiveness ratio (ICER). Sensitivity analyses were performed to examine the robustness of the base-case results.

**Results:**

In the base-case analysis, mHealth-based care gained higher QALYs of 0.0730 with an incurred cost of US $1090. Using US $33,438 per QALY (three times the gross domestic product) as the willingness-to-pay threshold, mHealth-based care was cost-effective, with an ICER of US $14,936 per QALY. In one-way sensitivity analysis, no influential factor with a threshold value was identified. In probabilistic sensitivity analysis, mHealth-based care was accepted as cost-effective in 92.33% of 10,000 iterations.

**Conclusions:**

This study assessed the expected cost-effectiveness of applying mHealth-based integrated care for AF according to a model-based health economic evaluation. The exploration suggested the potential cost-effective use of mHealth apps in streamlining and integrating care via the Atrial fibrillation Better Care (ABC) pathway for AF in China. Future economic evaluation alongside randomized clinical trials is highly warranted to verify the suggestion and investigate affecting factors such as geographical variations in patient characteristics, identification of subgroups, and constraints on local implementation.

## Introduction

Atrial fibrillation (AF) is the most frequent cardiac rhythm disorder. Approximately 2% of the population is affected by AF in European and North American countries, with the prevalence varying from 0.1% to 7.2% across different regions [1,2]. AF is less common in China than in Western countries, with a prevalence of 0.71% among adults above 35 years old [3]. Yet, the prevalence is higher than previously reported (0.65% for the population above 30 years) a decade ago, which is likely due to low awareness and huge treatment gaps [3-6]. According to a recent nationwide survey, the estimated number of patients with AF in China was 7.9 million [5]. For adults above 55 years of age, the lifetime risk of developing AF was approximately 1 in 5.3 [7]. Driven by a growing and aging population, the prevalence is predicted to at least double in the next 30 years [8].

The increasing prevalence of AF is associated with more health care utilization and health care expenditure. Stroke is the most common subsequent outcome, and patients with AF have a 5-fold increased risk of stroke [9]. Stroke-related care is costly, with incurred hospitalization costs of US $3000 to US $10,000 per patient in China [10]. Stroke patients with AF were more likely to have comorbidities, and their stroke-related costs were 20% more costly than those of stroke patients without AF [11,12]. Stroke prevention, proactive management of comorbidities, and lifestyle changes are essential priorities in AF care. Therefore, the Atrial fibrillation Better Care (ABC) pathway, a holistic and integrated approach, has been proposed to monitor anticoagulant therapy and manage the cardiovascular risks of patients with AF [13].

Adoption of the ABC pathway was found to be effective in reducing clinical adverse events and related health care costs in the Atherosclerosis in Atrial Fibrillation trial [14]. Another study applying the ABC pathway in the mobile health (mHealth) context also supported the favored effectiveness of integrated care for AF [15]. In this study, 1261 subjects who received mHealth technology–supported care were followed up over 1 year and had a lower risk of composite outcomes of “ischemic stroke (IS)/systemic thromboembolism, death, and rehospitalization” compared with that of their counterparts receiving usual care. mHealth-based care has been demonstrated to be cost-effective in managing diabetes, hypertension, and heart failure; however, the health economic impact of mHealth for patients with AF remains unknown [16-18].

A Markov model is a well-established analytic framework in the economic evaluation of health care interventions by using mutually exclusive disease states to represent all possible consequences. The purpose of this study was to perform a cost-effectiveness analysis via a Markov model to examine the clinical and health economic outcomes of mHealth-based integrated care for patients with AF in China.

## Methods

### Model Structure

**Figure 1:**
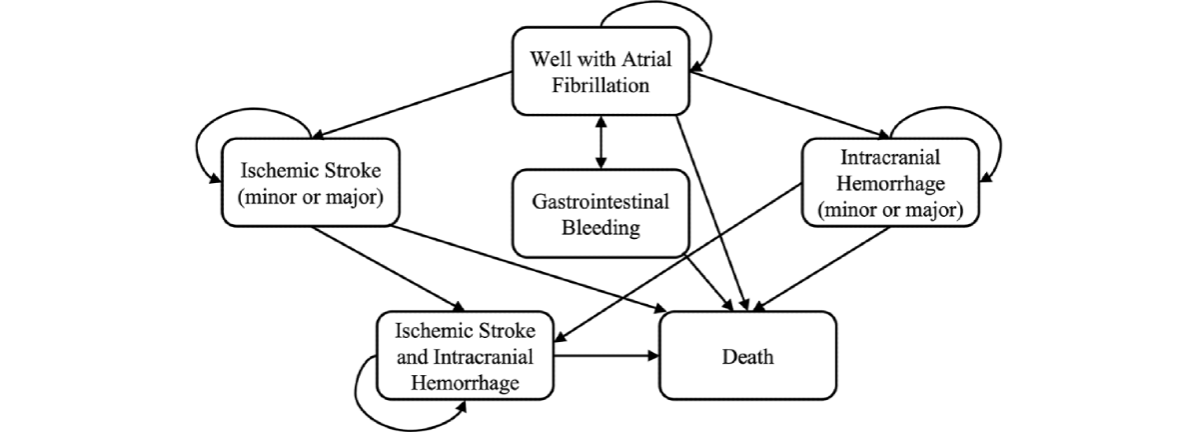
Schematic representation of the Markov model.

A Markov model was developed to evaluate the cost-effectiveness of mHealth-based care integrating the ABC pathway for patients with AF from the perspective of the public health care provider in China (Figure 1). The cycle length for the model was monthly cycles with a 30-year time frame to estimate the long-term effects. The baseline model population consisted of patients with AF with a mean age of 68 years and a median CHA_2_DS_2_-VASc (congestive heart failure, hypertension, age≥75 years [doubled], diabetes, stroke/transient ischemic attack/thromboembolism [doubled], vascular disease [prior myocardial infarction, peripheral artery disease, or aortic plaque], age 65 years, sex category [female]) score of 3 [15]. The patient characteristics were derived from the Mobile Atrial Fibrillation App (mAFA)-II trial, a cluster-randomized trial examining the first mHealth technology–based program for following patients with AF based on the ABC pathway in China. The clinical effectiveness from the mAFA-II trial was applied as the key model input after entering a hypothetical cohort of AF patients to the model. The model structure was adapted from the study published by Shah et al [19], where a Markov model was developed to model the prognosis of patients with AF using similar events of interest regarding stroke adopted in this study. Specifically, the strategies examined were mHealth-based care and usual care for patients with AF. The outcome measures were direct medical cost, quality-adjusted life years (QALYs), and incremental cost-effectiveness ratio (ICER).

The model consisted of the following Markov states: well with AF, minor and major IS, minor and major intracranial hemorrhage (ICH), IS and ICH, and death, with a temporary health state of gastrointestinal bleeding (GIB). With either mHealth-based care or usual care, all patients entered the model at the health state of being well and transitioned to another health state in the next cycle. The events of IS and ICH could each be of two types: minor and major. Once ICH or GIB occurred, patients would discontinue the anticoagulant therapy and switch to aspirin for the remaining life years. After stroke, patients might experience recurrent events. They might stay in the same health state or proceed to the health state of “IS and ICH.” Consistent with Shah et al [19], we assumed patients would advance to corresponding major events after two minor neurological events and that two major events would lead to death. Patients, in whichever state, could proceed to death. Patients in the arm of mHealth-based care might not be adherent to the mobile technology and would receive the same intervention as patients with usual care. Once the event occurred, these patients would receive mHealth-based care again in the next cycle.

Patients with usual care would receive treatment following the Chinese Stroke Association guideline [20]. Patients with mHealth-based care would receive integrated management based on the ABC pathway. The ABC pathway components consisted of Avoid Stroke with Anticoagulation (A), Better Symptom Management (B), and Cardiovascular Risk and Comorbidity Management (C). Specifically, patients allocated to the mHealth-based care would install a mobile app connected to a local public hospital via the internet. For Avoid Stroke with Anticoagulation (A), the laboratory results (eg, international normalized ratio, renal/hepatic function) tested in hospitals could be uploaded in the mobile app. The validated algorithm with confirmation from doctors would provide patients with data on anticoagulant monitoring, bleeding risk assessment, and guideline-based dosage adjustment. For Better Symptom Management (B), patients would receive a photoplethysmography smart device connected to the mobile app. They could send the cardiac rhythm monitoring data along with other symptoms such as headache and chest pain to the on-call doctors by the in-built communication function. Advice on rate or rhythm control would be given in a timely manner. Once a patient’s condition deteriorates, the management would be escalated to inpatient care. For Cardiovascular Risk and Comorbidity Management (C), patients’ comorbidities (eg, blood pressure) would be monitored with treatment optimized for blood pressure <140/85 mmHg. Lifestyle recommendations would also be given by educational articles, videos, and game-playing in the mobile app.

### Clinical Probabilities

All model inputs are listed in Table 1. The clinical inputs were retrieved from the published reports written in English, identified from a literature search on Medline throughout 2000-2021. Epidemiology or disease burden in the Chinese population, randomized clinical trials, and meta-analyses were preferred sources for clinical model inputs.

The probabilities of clinical events (IS, ICH, and GIB) after AF in usual care, the effectiveness (measured in hazard ratios) of mHealth-based care (vs usual care), and the compliance of mHealth support were retrieved from the mAFA-II trial (N=2473 patients) [15]. In the trial, a structured program of holistic and integrated care based on the ABC pathway via a mobile app was compared to usual care in patients with AF. The 18-month incidence of IS (4.12%), ICH (0.41%), and GIB (0.58%) in the usual-care arm was converted into the monthly probability (0.244%, 0.024%, and 0.034%, respectively) using the equation p=1–e^–rt^ (where p is probability, r is the event rate, and t is the cycle length) from the practical guide for Markov models [21]. The hazard ratio (mHealth-based care vs usual care) was 0.11 (95% CI 0.05-0.27, *P*<.001) for IS and 0.37 (95% CI 0.20-0.70, *P*=.002) for GIB [15]. No event of ICH was reported in the mHealth-based care group over 1-year follow-up. Considering the feasibility in long-term practice, the change of mHealth-based care on ICH incidence was assumed to be 0.5 in the base case, with a range of 0-1. The incidence of IS, ICH, and GIB for patients managed via mHealth technology was approximated by the incidence in usual care and the corresponding hazard ratio, as recommended by the Guide to the Methods of Technology Appraisals 2013 [22]. The proportion of minor, major, and fatal IS/ICH was estimated at 51.6%/49.5%, 40.2%/14.1%, and 8.2%/36.4%, respectively, from a study comparing outcomes of five oral anticoagulants for stroke prevention [19]. The incidences of recurrence and proportion of types of events (ICH or IS) were estimated from a 9-year community-based study of 0.5 million Chinese adults assessing the recurrent events after the first incident stroke [23]. The reported 5-year recurrence was 41% for IS (91% IS and 9% ICH) and 44% for ICH (44% IS and 56% ICH). Using the equation p=1–e^–rt^, the monthly probability was approximated to be 0.68% for IS and 0.73% for ICH [22]. The age-specific mortality rates were retrieved from a nationwide survey conducted in China [24]. The relative risk of death (with vs without GIB) was 3.5, with a range of 2.8 to 4.2 [25]. The mortality of GIB was calculated using age-specific mortality and the relative risk [22]. A population-based prospective study in an elderly (≥60 years) Chinese population identified an increased risk of all-cause mortality for AF, with a hazard ratio of 1.87 (95% CI 1.09-3.20, *P*=.02) [26]. The mortality of AF was estimated by the hazard ratio and age-specific mortality [22].

**Table 1:** Model inputs of clinical probabilities, utilities, and costs.

Variables				Base-case input (range)		Distribution		Reference
**Clinical variables**								
	**Probability of an event in usual care (monthly)**							
		IS^a^	0.244 (0.20-0.29)		Beta		Guo et al [15]	
		ICH^b^	0.024 (0.02-0.03)		Beta		Guo et al [15]	
		GIB^c^	0.034 (0.03-0.04)		Beta		Guo et al [15]	
	**Hazard ratio of events (mobile health–based care vs usual care)**							
		IS	0.11 (0.05-0.27)		Lognormal		Guo et al [15]	
		ICH	0.5 (0-1)		Triangular		Guo et al [15], assumption	
		GIB	0.37 (0.2-0.7)		Lognormal		Guo et al [15]	
	Compliance of mobile health–based case, %			70.8 (50-100)		Beta		Guo et al [15]
	**Proportion of events, %**							
		IS minor	51.6 (43.9-55.8)		Dirichlet		Shah et al [19]	
		IS major	40.2 (40.2-41.7)		Dirichlet		Shah et al [19]	
		IS fatal	8.2 (2.5-16.3)		Dirichlet		Shah et al [19]	
		ICH minor	49.5 (33-63)		Dirichlet		Shah et al [19]	
		ICH major	14.1 (9-21.4)		Dirichlet		Shah et al [19]	
		ICH fatal	36.4 (15.6-58.0)		Dirichlet		Shah et al [19]	
	**Probability of stroke recurrence (monthly)**							
		IS	0.68 (0.68-0.70)		Beta		Chen et al [23]	
		ICH	0.73 (0.70-0.76)		Beta		Chen et al [23]	
	**Proportion of recurrent events, %**							
		IS after IS	91 (73-100)		Beta		Chen et al [23]	
		ICH after IS	9 (0-27)		Beta		Chen et al [23]	
		IS after ICH	44 (33-55)		Beta		Chen et al [23]	
		ICH after ICH	56 (45-67)		Beta		Chen et al [23]	
	**Age-specific (years) mortality (monthly), %**							
		65-69	0.10 (0.08-0.12)		Triangular		National Bureau of Statistics [24]	
		70-74	0.26 (0.20-0.31)		Triangular		National Bureau of Statistics [24]	
		75-79	0.41 (0.33-0.50)		Triangular		National Bureau of Statistics [24]	
		80-84	0.71 (0.57-0.85)		Triangular		National Bureau of Statistics [24]	
		85-89	1.06 (0.85-1.27)		Triangular		National Bureau of Statistics [24]	
		90-94	1.59 (1.27-1.91)		Triangular		National Bureau of Statistics [24]	
		>95	1.81 (1.45-2.17)		Triangular		National Bureau of Statistics [24]	
**Utilities**								
	Event-free AF^d^		0.9 (0.8-1)		Uniform		Shah et al [19]	
	Minor IS		0.75 (0.6-0.92)		Uniform		Shah et al [19]	
	Major IS		0.39 (0.31-0.47)		Uniform		Shah et al [19]	
	Minor ICH		0.75 (0.6-0.92)		Uniform		Shah et al [19]	
	Major ICH		0.39 (0.31-0.47)		Uniform		Shah et al [19]	
	Utility decrement of GIB		0.16 (0.13-0.19)		Uniform		Shah et al [19]	
**Costs (US $)**								
	**Event-related costs (per episode)**							
		Minor IS	3277 (2622-3932)		Lognormal		Chang et al [10]	
		Major IS	6676 (5341-8012)		Lognormal		Chang et al [10]	
		Minor ICH	5284 (4227-6340)		Lognormal		Chang et al [10]	
		Major ICH	10567 (8454-12,680)		Lognormal		Chang et al [10]	
		GIB	3443 (2754-4131)		Lognormal		Chang et al [10]	
		All-cause death	5849 (4679-7019)		Lognormal		Chang et al [10]	
	**Follow-up cost (per month)**							
		Anticoagulation therapy	249 (199-299)		Lognormal		MENET [27]	
		Minor IS	328 (262-393)		Lognormal		Experts’ opinion	
		Major IS	668 (534-801)		Lognormal		Experts’ opinion	
		Minor ICH	528 (422-634)		Lognormal		Experts’ opinion	
		Major ICH	1057 (845-1268)		Lognormal		Experts’ opinion	
	Cost of site implementation per patient (one-time cost)		80 (64-96)		Lognormal		Boodoo et al [28]	
	Cost of managing per month		15 (12-18)		Lognormal		Zhang and Liu [29]	

^a^IS: ischemic stroke.

^b^ICH: intracranial hemorrhage.

^c^GIB: gastrointestinal bleeding.

^d^AF: atrial fibrillation.

### Utility and Utility Adjustment

Literature-based utilities were assigned to each health state to calculate QALYs (Table 1) [19]. Major or minor neurological events were associated with permanent disutility. Temporary disutility was applied for GIB with a duration of 14 days. The expected QALYs were estimated by the utility in each state and the time spent. The QALYs gained were discounted at 3.5% per annum, as recommended by the Guide to the Methods of Technology Appraisals 2013 [22].

### Resource Use and Costs

All costs were considered from a public health care provider’s perspective in China and only direct medical costs were included (Table 1). All cost inputs were retrieved from public data. The assumption (if necessary) was made in consultation with local experts, which was considered a legitimate source of information for decision-analytic modeling [30]. The event costs of IS (minor or major), ICH (minor or major), and GIB were estimated from the in-hospital direct costs for thromboembolism and bleeding in Chinese patients with AF, which were collected from seven representative tertiary referral hospitals and three secondary-care hospitals [10]. The cost of all-cause death was approximated by the mean of the event-related cost. The cost of medication was estimated by the frequency and the median cost of anticoagulants in the Menet database [27]. Since limited data were available on the cost of postevent follow-up, the cost was assumed to be 1/10 the event cost based on local cardiac specialists’ advice. The one-off charge for site implementation, including a wristband-type wireless photoplethysmographic device, was estimated from the reported cost of a smartphone-based system for heart failure [28]. We assumed that the clinical doctor in charge would spend 1 hour monitoring a patient every month. The monthly cost for subscribing to the mHealth-based service was estimated from physicians’ hourly rate and the time spent on the service [29]. All costs were adjusted to the year 2021, with an annual discount rate of 3.5%, based on the recommendations from the Guide to the Methods of Technology Appraisals 2013 [22].

### Analytic Methods

All model parameters were used to generate the cohort model. Model validation was performed by comparing the estimated incidence of events to the reported outcomes in the mAFA-II trial and comparing the simulated 5-year survival rate to that reported for the Chinese elderly population with AF [31]. The direct medical costs and QALYs of each comparator were calculated over a 30-year time horizon. ICERs were estimated and compared against the willingness-to-pay (WTP) threshold. The WTP threshold was defined as three times the gross domestic product per capita in China, according to the World Health Organization recommendation [32]. The gross domestic product per capita was US $11,146 (US $1=RMB 6.5); thus, the WTP threshold was US $33,438 per QALY [33].

One-way sensitivity analysis was performed to assess the robustness of the base-case results. The literature-available ranges were adopted (Table 1). Otherwise, ±20% of the base-case values were used to examine the impact of parameters on the ICER. Parameter uncertainty was determined using 10,000 Monte Carlo simulations by varying all inputs simultaneously with random draws from each specified distribution. The results of probabilistic sensitivity analysis are presented in a scatterplot in the form of incremental costs against incremental QALYs. The probability of each strategy to be preferred was determined in the cost-effectiveness acceptability curve over US $0-50,000 per QALY. All analyses were performed using Excel 2016 software (Microsoft Corp).

## Results

### Model Validation

To examine the predictive validity of the model, the simulated event rates (IS, ICH, and GIB) in usual care and mHealth-based care were compared to the findings reported in the mAFA-II trial [15]. As shown in Table 2, all simulated events in both arms were within 10% of relative difference when compared with the reported data from the mAFA-II trial. The simulated 5-year survival rate determined by the model (73.5%) was also compared to that reported (68.9%) for the Chinese elderly population with AF (CHA_2_DS_2_-VASc score of 3), and the relative difference was found to be 6.67% [31].

**Table 2:** Model validation.

Variable	Usual care (median follow-up 546 days)				mHealth^a^-based care (median follow-up 701 days)		
	Trial	Model	Difference	Trial		Model	Difference
IS^b^	4.12%	3.86%	–6.31%	0.48%		0.43%	–10.42%
ICH^c^	0.41%	0.40%	–2.44%	—^d^		—	—
GIB^e^	0.58%	0.55%	–5.17%	0.40%		0.39%	–2.50%

^a^mHealth: mobile health.

^b^IS: ischemic stroke.

^c^ICH: intracranial hemorrhage.

^d^The incidence of ICH in the trial was reported to be 0 within follow-up. The model simulated the long-term impacts and assumed that the relative risk of mHealth-based care (vs usual care) regarding ICH was 0.5, with a range of 0-1. Therefore, the difference in the ICH incidence is not presented.

^e^GIB: gastrointestinal bleeding.

### Base-Case Analysis

Over a 30-year time horizon, the total costs of mHealth-based care and usual care were US $35,691 and US $34,601, respectively. The expected QALY gain was 7.2749 for mHealth-based care and 7.2019 for usual care. Compared with usual care, mHealth-based care gained additional QALYs of 0.0730 with an incurred cost of US $1090. The ICER was US $14,936 per QALY, which was below the WTP threshold of US $33,438 per QALY. These results indicated mHealth-based care as a cost-effective strategy in the base-case analysis.

### Sensitivity Analyses

**Figure 2:**
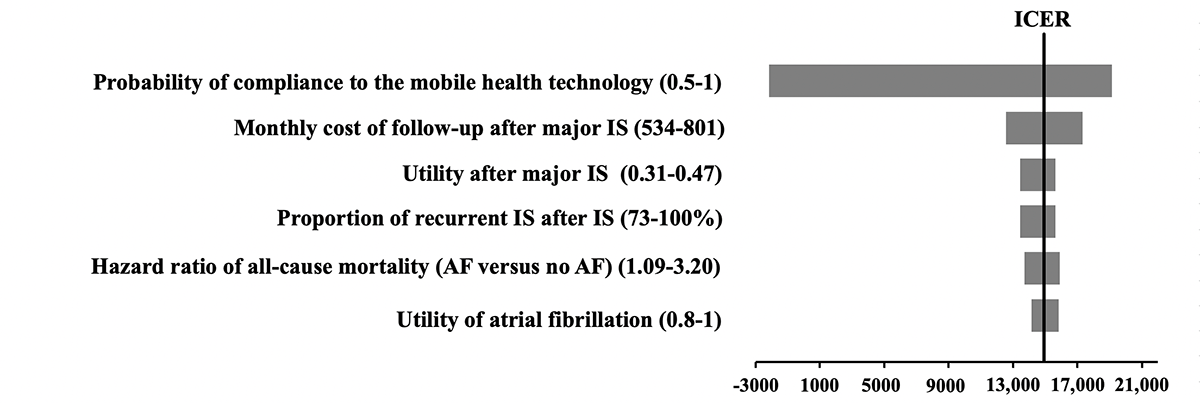
Tornado diagram of one-way sensitivity analysis summarizing the effect of parameters on the ICER. ICER: incremental cost-effectiveness ratio; IS: ischemic stroke; AF: atrial fibrillation.

The ICERs of mHealth-based care were all below the WTP threshold throughout the one-way variation. No parameter with a threshold value was found (Figure 2). The analysis showed that the model results were most sensitive to compliance to mHealth-based care, the monthly cost of follow-up after major IS, utility after major IS, the proportion of recurrent IS after IS, the hazard ratio of all-cause mortality (AF vs no AF), and utility of AF. As the compliance of mHealth-based care had the most significant impact on the ICER, an extended one-way sensitivity analysis was performed. Once the probability of compliance to the mobile technology exceeded 99%, mHealth-based care was a cost-saving option with QALYs gained when compared with usual care.

**Figure 3:**
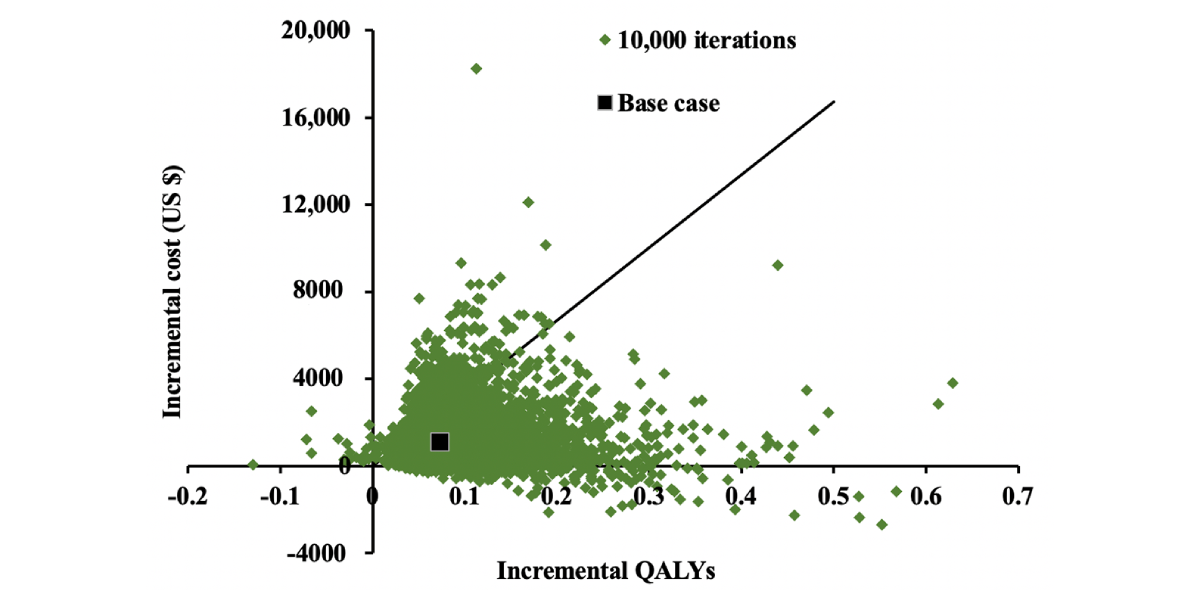
Incremental cost-effectiveness scatterplot: probabilistic sensitivity analysis for mobile health–based care versus usual care. QALYs: quality-adjusted life-years.

The Monto Carlo simulations of incremental costs versus incremental QALYs gained by mHealth-based care are shown in Figure 3. The mHealth-based care gained average QALYs of 0.0842 (95% CI 0.0832-0.0851, *P*<.001), with a mean additional cost of US $1053 (95% CI US $1033-1073, *P*<.001). Of 10,000 iterations, mHealth-based care gained higher QALYs at a higher cost with the ICER below the WTP threshold 89.44% of the time. The probability of mHealth-based care being effective in QALYs gain with cost savings was 2.89%.

**Figure 4:**
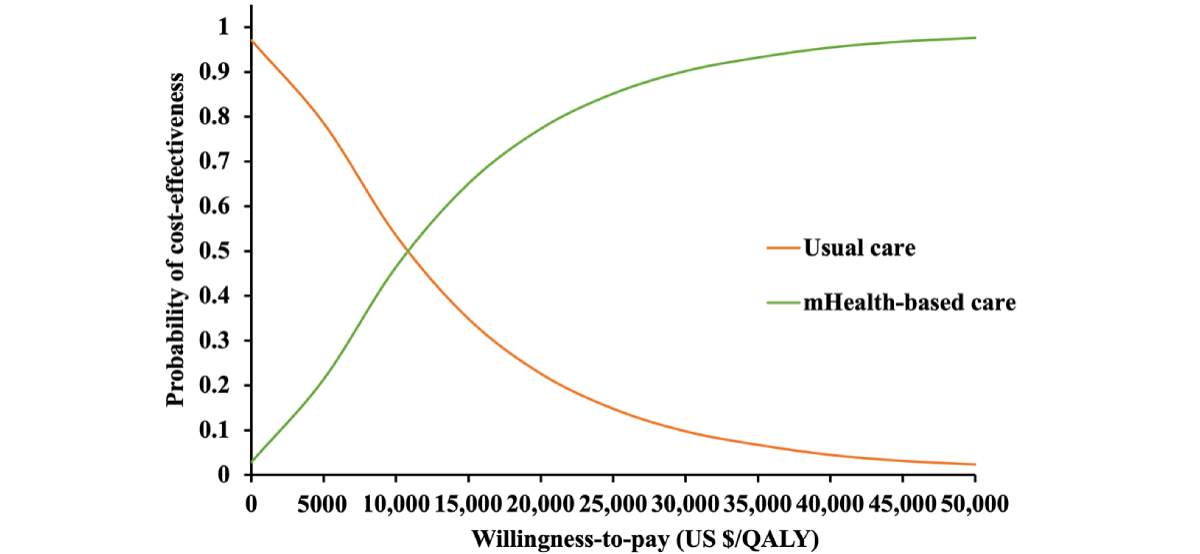
Cost-effectiveness acceptability curve. mHealth: mobile health. QALY: quality-adjusted life year.

The probability of each comparator being preferred as cost-effective is shown in the acceptability curve of Figure 4. The mHealth-based care and usual care shared the same probability (50%) of being cost-effective at the WTP threshold of US $10,699 per QALY. mHealth-based care was accepted to be cost-effective 92.33% of the time at the WTP threshold of US $33,438 per QALY.

## Discussion

### Principal Results

This is the first cost-effectiveness analysis examining mHealth-based integrated care using the ABC pathway to manage patients with AF. Compared to usual care, mHealth-based care was cost-effective from the public health care provider in China, with an ICER (US $14,936 per QALY) below the WTP threshold (US $33,438 per QALY). No parameter varying the ICER with a threshold value was found in one-way sensitivity analysis, indicating the robustness of the base-case results. In probabilistic sensitivity analysis, the probability of mHealth-based care being preferred was high throughout the WTP threshold variation, which further supported the cost-effective application of managing patients with AF via mHealth technology. To our best knowledge, no cost-effectiveness analysis has previously been performed to examine the application of mHealth technology in care after an AF diagnosis, although such an analysis has been performed for screening [34]. Our findings are in line with the results of previous cost-effectiveness analyses investigating mHealth tools for other cardiovascular diseases, indicating the cost-effective use of the mHealth support system [16-18]. The small improvement in QALYs by the mHealth-based care for AF, similar to other digital health technologies, may be driven by a small, estimated gain in survival and reflect the indirect effects of the technologies on mortality.

A recent community-based multicenter study investigating the prevalence of untreated AF found a noticeable treatment gap in urban China. Only 20.3% (28/138) of patients with AF qualifying for guideline-recommended anticoagulant therapy commenced the treatment [6]. The undertreated AF resulted from patients’ preference to attend local community centers over specialist clinics, community health center physicians’ lack of knowledge regarding evidence-based management (only antiplatelet drugs and traditional Chinese medicine prescribed), and specialists’ low adherence to AF guidelines. The condition is likely to be worse in rural China owing to the lower awareness in rural residents than in urban areas [6]. To optimize the management of patients with AF, a mobile technology–supported program adopting the ABC pathway was initiated in China [15,35]. The program encompassed guideline-adherent recommendation and monitoring on anticoagulants, patient-centered symptom-directed decisions for rate or rhythm control, and comorbidity management. The results showed that mHealth-based care was associated with improved patient outcomes: better rate/rhythm control; increased use of anticoagulants; and reduced composite outcomes of IS/systemic thromboembolism, death, and rehospitalization.

As China’s population is aging, there is a shift in the disease burden to chronic noncommunicable diseases. The Chinese government is actively seeking ways to reduce the health care expenditure on chronic diseases. The delivery of quality health services in a cost-effective manner is the key direction [36]. In this landscape, mHealth apps offer unique opportunities for improving the quality of care while reducing the cost of care by outsourcing the proactive patient monitoring to a clinically validated algorithm, enabling early diagnosis and intervention to patients, and saving clinicians’ time for more urgent cases. Due to a lack of economic evidence, the innovative use of mobile technology has not yet been adequately integrated into the health care system. Therefore, cost-effective assessment of mHealth-based care is the essential process of considering this new technology in China. Our study demonstrated that using mobile technology to streamline and integrate care for patients with AF is likely to be cost-effective from the perspective of public health care providers in China. A recent study suggested patients’ problems of seeking routine care under the context of the COVID-19 pandemic and increasing use of internet-based medical services in China [37,38]. In this regard, internet-based interventions with cost-effectiveness, such as mHealth-based care for AF patients, should be considered part of routine care for chronic diseases.

mHealth-based care involves patients’ engagement more than conventional care. Patients’ compliance and persistence are significant barriers and challenges for advancing these new technologies [39]. The realization of the benefits of mHealth technology will only occur when high compliance is achieved. Key factors to improve patients’ compliance encompass user training, active human support, and telehealth implementation style [40]. To examine the compliance on the findings, an extended one-way sensitivity analysis varying the parameter from 0%-100% (base-case value 70.8%) was performed. Once full adherence (≥99%) is achieved, the mHealth-based care would be a cost-saving option with QALY gains. Compliance is a nontransferrable parameter among different health care systems. Local patients’ willingness to uptake and adhere to the technology should be evaluated in a pilot study before incorporating mHealth support into AF care.

### Limitations

The results of this model should be interpreted while considering the following limitations. First, the model was developed based on a cluster-randomized clinical trial (mAFA-II trial), which studied an adult population diagnosed with AF who were followed up over 1 year. The baseline characteristics were adopted for the model population [15]. In the trial, the intervention group consisted of 1261 subjects (mean age 67 years, median CHA_2_DS_2_-VASc score 3, 34.1% women) and 1212 subjects (mean age 70 years, median CHA_2_DS_2_-VASc score 3, 42.1% women). A community-based survey of 47,841 adults (aged ≥45 years) in seven geographic regions of China showed the characteristic of confirmed AF patients (mean age 67.6 years, CHA_2_DS_2_-VASc score of 2.13 for men; mean age 66.6 years, CHA_2_DS_2_-VASc score of 3.08 for women), which indicated the similarity of Chinese AF patients and the model population [5]. However, fewer women were identified in the mAFA-II trial than reported in the community-based survey (54.7%). This likely resulted from the selection bias with more male, younger subjects included during AF screening phases, which limited the generalizability of the results [15].

Second, the effectiveness estimates, both probabilities and hazard ratios, were approximated from a follow-up much shorter than the lifetime horizon and used to examine the long-term effects of mHealth technology. To examine the model’s predictive validity, the simulated events were compared to rates reported in the trial, and the 5-year survival rate was also compared to that reported for the Chinese elderly population with AF. The model development, data conversion, and approximation followed the practical guide for Markov models and the guide to the methods of technology appraisal 2013 [21,22]. The model demonstrated accuracy with acceptable differences between simulated and reported rates. The higher 5-year survival rate generated in the present model was likely due to the model population being less elderly and having fewer comorbidities compared to patient characteristics in the cohort study [31]. For a more precise estimation of the cost-effectiveness over a lifetime scale, a clinical trial investigating the long-term effectiveness of mHealth-based care is highly warranted.

Third, this is a model-based health economic evaluation using model inputs from multiple sources with similar patient characteristics as the model population. Nevertheless, the data availability partially limited the data retrieval and the generalizability of the results to a large population with different region-specific patient characteristics in China. The variation was considered in one-way and probabilistic sensitivity analyses using the reported ranges or 20% of the base-case values. No threshold value was identified in one-way sensitivity analysis, and a strong likelihood of being cost-effective in probabilistic sensitivity analysis supported the applicability of the results. Future studies should investigate the use of mHealth technology for AF in randomized clinical trials considering geographical variations in patient characteristics, identification of subgroups, and constraints on local implementation, along with a trial-based economic evaluation assessing the incremental cost-effectiveness of mHealth tools.

Fourth, the cost inputs were retrieved from multiple sources according to data availability, including expert opinions on the monthly cost of postevent follow-up due to limited data. No identified threshold value of cost inputs indicated the robustness of the base-case result. Future studies investigating the cost of postevent follow-up in Chinese patients with stroke are warranted.

### Conclusion

This study assessed the expected cost-effectiveness of applying mHealth-based integrated care for AF by a model-based health economic evaluation. The exploration suggested the potential cost-effective use of mHealth apps in streamlining and integrating care via the ABC pathway for AF in China. Future economic evaluation alongside randomized clinical trials is highly warranted to verify the suggestion and investigate affecting factors such as geographical variations in patient characteristics, identification of subgroups, and constraints on local implementation.

## Abbreviations

ABC: Atrial fibrillation Better Care
AF: atrial fibrillation
CHA_2_DS_2_-VASc: congestive heart failure, hypertension, age ≥75 years (doubled), diabetes, stroke/transient ischemic attack/thromboembolism (doubled), vascular disease (prior myocardial infarction, peripheral artery disease, or aortic plaque), age 65 years, sex category (female)
GIB: gastrointestinal bleeding
ICER: incremental cost-effective ratio
ICH: intracranial hemorrhage
IS: ischemic stroke
mAFA-II: Mobile Atrial Fibrillation App trial
mHealth: mobile health
QALY: quality-adjusted life year
WTP: willingness to pay
